# Intercellular trafficking via plasmodesmata: molecular layers of complexity

**DOI:** 10.1007/s00018-020-03622-8

**Published:** 2020-09-12

**Authors:** Ziqiang Patrick Li, Andrea Paterlini, Marie Glavier, Emmanuelle M. Bayer

**Affiliations:** 1 Univ. Bordeaux, CNRS, Laboratoire de Biogenèse Membranaire, UMR 5200, F-33140 Villenave d’Ornon, France; 2 Sainsbury Laboratory, University of Cambridge, Cambridge, UK

**Keywords:** Plants, Cell–cell communication, Plasmodesmata, ER–PM contacts, Nanodomains, Cell wall

## Abstract

Plasmodesmata are intercellular pores connecting together most plant cells. These structures consist of a central constricted form of the endoplasmic reticulum, encircled by some cytoplasmic space, in turn delimited by the plasma membrane, itself ultimately surrounded by the cell wall. The presence and structure of plasmodesmata create multiple routes for intercellular trafficking of a large spectrum of molecules (encompassing RNAs, proteins, hormones and metabolites) and also enable local signalling events. Movement across plasmodesmata is finely controlled in order to balance processes requiring communication with those necessitating symplastic isolation. Here, we describe the identities and roles of the molecular components (specific sets of lipids, proteins and wall polysaccharides) that shape and define plasmodesmata structural and functional domains. We highlight the extensive and dynamic interactions that exist between the plasma/endoplasmic reticulum membranes, cytoplasm and cell wall domains, binding them together to effectively define plasmodesmata shapes and purposes.

## Introduction

“I tried to explain as much as I could—Poppet says. I think I made an analogy about cake Well that must have worked—Widget says. Who doesn’t like a good cake analogy?”

E. Morgenstern—The Night Circus (2011).

Unicellular and multicellular organisms share—among other traits—the fundamental need for communication. This is not to be intended in its verbal connotation but rather as the diverse array of molecular mechanisms used to coordinate biological processes within and between organisms. A high order classification divides signalling into intracrine (happening within a cell), autocrine (secretion of molecules that act on the secreting cell itself), juxtacrine (between physically touching cells), paracrine (aimed at cells in the vicinity of the signalling source) and endocrine (the signal produced can travel to distant cells) (reviewed in [1]). This classification is more widely employed in animal research but we feel it similarly carries value for research in other organisms, albeit with conceptual adjustments for their specific biology.

**Fig. 1:**
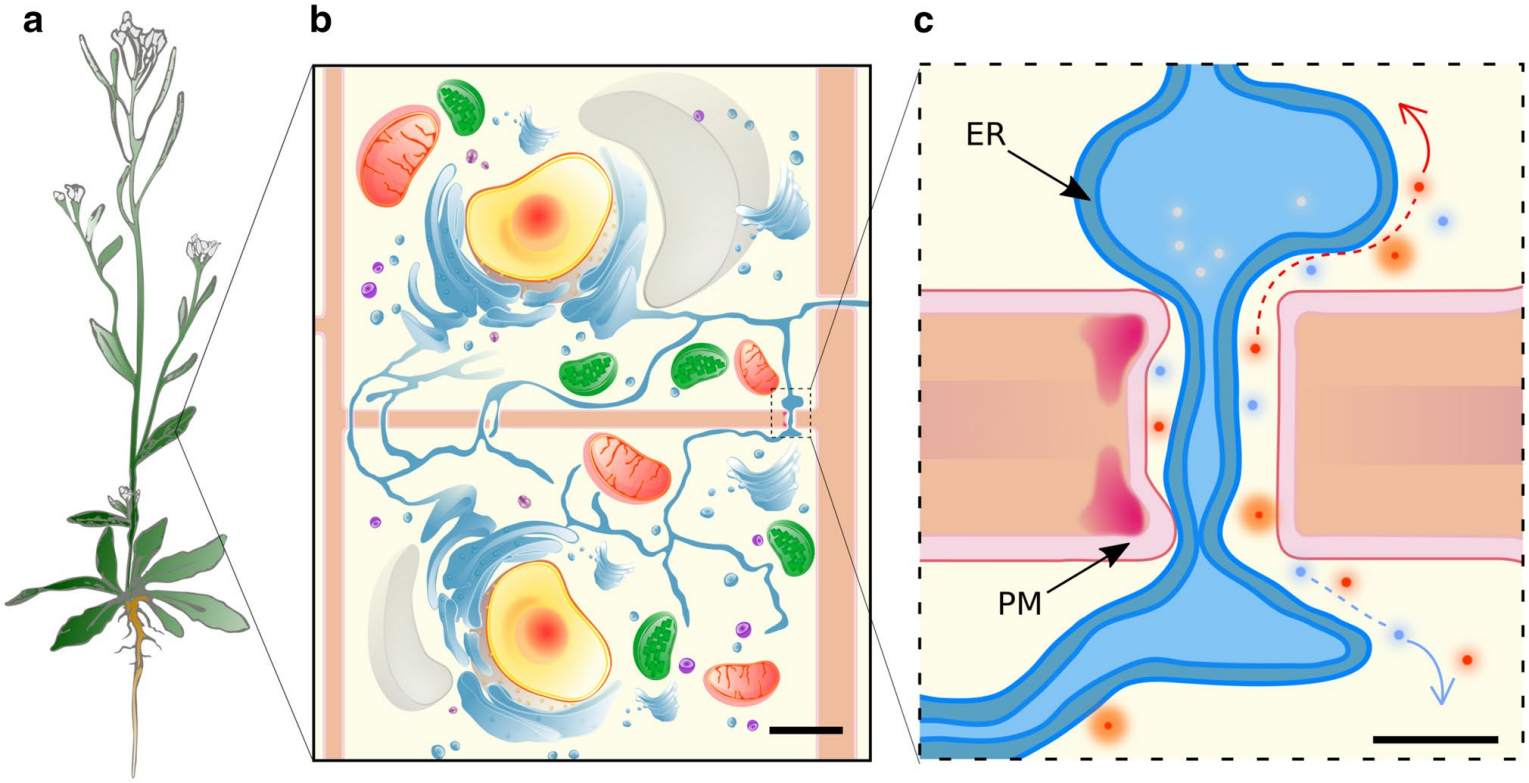
Whole organism to single-cell multiscale view, emphasising cell–cell connectivity via plasmodesmata (PD). **a** Schematic representation of *Arabidopsis thaliana* at flowering stage. **b** Two plant cells showing their cytoplasmic contents (nucleus in yellow, mitochondria in red, vacuole in grey, cell wall in coral, endoplasmic reticulum (ER) in blue, peroxisome in violet and ribosomes in purple, cytoplasm in light yellow, chloroplast in green) and displaying PD at their cell–cell interfaces. **c** PD are plasma membrane (PM; pink) lined, cell wall (coral) spanning pores that enable transport of molecules (red and light blue circles), mostly across the cytoplasmic sleeve (light yellow space). PD neck constriction via deposition of the wall polysaccharide callose (red) can reduce trafficking across the pores. The proposed model is through reduced ER–PM spacing (left side of panel **c**). Physical continuity of the endomembrane system (ER and PM) is also observable in panels **b** and **c** with the ER becoming highly constricted within PD and largely preventing lumenal transport of macromolecules. Examples of potential directional transports are shown by the coloured arrows. Differences in PD densities between cellular interfaces (basal vs lateral sides in panel **b**) are also represented. Scale bars: 50 μm in (**b**) and 50 nm in (**c**). Abbreviations: *ER*, endoplasmic reticulum. *PD,* plasmodesmata. *PM*, plasma membrane.

Our focus is more closely aligned with a type of juxtacrine (and also possibly aspects of intracrine, paracrine and endocrine as explained at various stages in this review) signalling as we study plasmodesmata (PD) pores that put in direct contact the cytoplasm of two neighbouring cells (Fig. 1). These should not be mistaken for passive channels as continuous and extensive regulation is operated upon them (reviewed in [2] in the context of horticultural applications). Direct cytosolic cell–cell signalling strategies are observed throughout the kingdom of life albeit with significant differences in their molecular composition and their mode of action. Septal junctions connecting filament-forming cyanobacteria were recently structurally resolved as a multimeric protein complex [3]. Gap junctions between animal cells are also known to be proteinaceous in nature (reviewed in [4]). Tunneling nanotubes that bridge neuronal cells are actin enriched membranous protrusions with open ends [5]. Conversely, in plants, PD include a continuous plasma membrane (PM) traversing the cell wall between neighbouring cells and a constricted form of the endoplasmic reticulum (ER), the desmotubule, spanning the pore in its center (reviewed in [6]) (Fig. 1c). At PD, the ER and PM are tethered together by protein elements, leaving a space, termed the cytoplasmic sleeve between the two. Unique to PD is, therefore, the duplex endomembrane continuity between cells, in addition to the cytoplasm one.

Together with other cell–cell communication mechanisms, PD play a central role in plant development and physiology. They enable metabolite fluxes between cells ([7] as an example in the context of plants with different photosynthetic strategies), they contribute to the distribution of key plant hormones involved in development ([8] as an example for auxin) and they control the movement of RNA/proteins acting as developmental regulators ([9] as an example in the context of plant stem cell maintenance). PD are also fundamental for long distance transport of resources and signals to distant organs, a function that might be reminiscent of endocrine signalling. PD influence the loading [10], translocation [11] and ultimate release of substances [12] from the phloem, the specialised conduit connecting distal organs within the plant.

PD provide four potential routes for intercellular trafficking: a main symplastic one across the cytoplasmic sleeve, two membrane ones along either the PM or the ER and a luminal one within the desmotubule. The cytoplasmic sleeve one has long been and still is regarded as the main route of transport for hydrophilic, soluble mobile factors (reviewed in [13, 14]). Molecules with strong hydrophobic properties (or with domains displaying such properties) can conversely in theory take advantage of the ER/PM surface route. These factors would be anchored in the membranes [15, 16]. Movement within the desmotubule lumen has been more controversial, being largely ruled out in face of the extreme constriction of the ER membranes at PD and evidence supporting a lack of movement of luminal marker [17–19]

Overall, in this review, we employ the analogy of a multi-layered cake to deliver a series of key messages regarding PD. In the same way as a cake is a mixture of different ingredients, PD are careful assemblages of selected molecular components. Such assemblage is not coincidental, but rather the result of evolutional pressures and selection [20]. We also point out that how the different components are mixed together and relate to each other is essential, bakery being considered a science of precision. Lastly, as elaborate cakes present multiple different tiers, PD also encompass multiple structural layers (ER, cytoplasmic sleeve, PM and cell wall) that are physically and functionally interconnected. However, the imagery of a baked cake should not provide a false static image of PD as these structures undergo extensive and dynamic remodelling. Overall, as all analogies, ours also carries points of strength and weakness, but it is primarily meant to convey some important concepts in an engaging manner.

## Warming the oven with some key concepts: control of PD symplastic conductivity

A lingering narrative in PD research seems to postulate an “open” resting status of PD. This status is extrapolated as the natural opposite of the observed cases when PD were actively “closed” in response to external clues. The cytosolic cell–cell continuity enabled by PD and the presence of a continuous symplastic space is for instance a double-edged sword when exploited by invading pathogenic organisms (reviewed in [21]). One of the physiological responses of the cells to such challenges (and similarly upon abiotic stresses) tends to be the closure of PD via over-accumulation of callose, a polysaccharide lining the cell walls of PD ([22, 23] as examples) (Fig. 1c). Callose can be detected via specific antibodies or stains ([24] as an example of both approaches). PD accumulating callose have been viewed as classical “closed” situations.

Boxing the conductivity of PD into resting (open) vs stressed/attacked (closed) statuses is, however, too simplistic and rather a more complex and nuanced picture exists. For instance, in an environmental context, conductivity was recently shown to vary during the day, being more prominent in presence of light and being conversely gated by circadian clock mechanisms at night [25]. Similarly, photoperiodic control of bud growth has also been related to PD closure, isolating the structure from growth signals specifically during winter [26]. In a developmental context, cotton fibres require a transient and reversible closure of PD and a switch to apoplastic loading specifically during their elongation phase, boosting osmotic and turgor pressure in the cell [27]. Similarly, during stages close to the final lateral root emergence, a transient isolation domain is established in the primordium [28]. Whether a PD is open or closed may therefore very much depend on when we ask this question. In addition to the timing, the specific location also seems to be a central aspect. For instance, lateral root primordia progression is accompanied by a temporally regulated PD closure in the specific tissues overlying the primordia [29]. It is still unclear why this induction occurs as it negatively correlates with lateral root emergence. However, if the degree of PD closure is quantitative rather than absolute, the mechanism described in [29] could be viewed as a point of regulation for the extent of root branching.

We should keep in mind that indeed cell–cell connectivity at a given interface does not depend on a single PD but rather on a population of them, adding further quantitative aspects ([7] as an example for metabolic fluxes in leaves). Intercellular transport will indeed depend on the overall status of a PD population, while individual PD may display different transport capacities. For instance, in addition to callose, the permeability of a PD also depends on its structure [30, 31] and perhaps on other yet unidentified factors. So far, the tools used in the field to assess symplastic transport include small injected/applied fluorescent dyes [32, 33], proteins expressed from endogenous tissues [34, 35] or bombarded on the same [36]. In most cases we only study an overall visible effect on the movement of molecules (very often non-native substances) across entire interfaces. Having access to the transport status of individual PD will be informative and will help, for instance, to understand whether fast coordination of responses occurs between PD. It is however experimentally challenging to address this particular point due to the nanoscopic size of individual PD, way beyond the light diffraction limit. In this regard, modelling approaches may provide an alternative way to appreciate whether or not the timing and speed of PD state changes is relevant for overall cell–cell interface connectivity.

In addition, the frequency and distribution of PD across tissues, even within different sides of the same cell, can be widely different ([37] as an example), adding extra levels of complexity to the system. This asymmetrical arrangement together with differing transport capacities of individual PD can result in directional transport across several cell layers by creating a channelling effect. Permeability differences have for instance been observed between lateral versus apico-basal interfaces in roots [35]. Functional impacts of such asymmetric flow were recently reported in [8, 38]. In an ideal situation, all of these parameters (distribution, density, structure, transport status) should be taken into account to accurately and comprehensively map the symplastic intercellular network. Combining experimental data and modelling approaches can, in principle, achieve this.

We already mentioned approaches to study interface permeabilities, to instead focus on the other parameters, different microscopy techniques can be informative. Confocal microscopy, for instance, addressed the occurrence of different types of PD at cellular interfaces of the leaf epidermis [39]; immunolocalization and scanning electron microscopy (EM) focused on PD densities and their surface occupancy at various leaf internal interfaces [7]; transmission electron microscopy on sections provided comprehensive PD maps for the root [40]; serial block EM was informative for PD densities at root vascular interfaces [11, 37] and electron tomography resolved the fine structures of PD in root cap and vascular cells [37, 41]. The spatial distribution of PD can be extracted from these datasets [42, 43] as it was shown to have again impacts on flow between cells, according to computational models (see in [44]). Temporal changes in PD frequencies and arrangements have also been uncovered with such techniques ([11, 40, 43] as examples).

The concept of PD “openness”/”closure” is also itself relative as it varies according to the substance being discussed. Different levels of permeability across PD depend on the shape, size and possibly electrostatic charge of the molecules attempting to cross. This combination of factors is routinely defined as the size exclusion limit of PD (SEL) [45, 46]. This differential permeability is not surprising. Indeed having a tight control on the movement of proteins that carry developmental programmes (via PD and other intracellular mechanisms) might be essential to maintain cell identity despite abundant connections ([47] using the SHORT ROOT (SHR) transcription factor as an example).

Based on traffic models, we can also categorize molecules into two types, those whose intercellular movement is non-targeted and those for which it is. The non-targeted molecules follow SEL requirements and are assumed to pass through PD cytoplasmic sleeve (reviewed in [48]). Both diffusion and advection might drive non-targeted molecular flow across PD [49]. The former is caused by concentration differences between cells for a given solute. This type of transport is directional: from region of high concentration to region of low concentration. However, this only applies to the specific solute displaying the concentration difference ([49]). Advective movement instead refers to mechanical transport through bulk motion (reviewed in [50]). Examples of this are pressure driven bulk flow in the phloem (reviewed in [50]) or cytoplasmic streaming (reviewed in [51]). In that case, all substances would be dragged along with the water flux, which sets the direction of transport. Evidence of transport unidirectionality indeed exists from trichome studies [52] and batch unloading in the root [12]. Additional, yet unexplored biophysical processes may also influence intercellular movement. For instance, surface fluctuation can modify the transport capacity of nanochannels by modifying diffusion and advection locally [53]. In all scenarios, PD geometry (such as pressure of a desmotubule, central cavity, constricted neck) but also internal membrane electrostatics are expected to be determinant. The relative contritubion of these different transport processes is then likely to depend on the cellular and environmental context.

In contrast to non-targeted transport, a number of plant native mobile factors have been shown to gate PD and modify SEL to facilitate their own transport across the cell border (reviewed in [13]). Non-native proteins produced by invading plant viruses (reviewed in [54], fungi [55] and bacteria [56] similarly exploit these gating strategies to enable spread of the pathogens, which would be normally impaired at PD resting state ([57] as an example of compromised movement of a virus lacking a functioning movement protein). The actual mechanisms of targeted movement, which can occur with or without basal SEL modification, most likely relates to interactions with local PD factors ([58] as an example). Additional or alternative modification of the mobile protein/mRNA might be necessary [59, 60].

However, it is also important to point out that PD are not isolated cellular structures but rather part of the broader organellar environment of the cell. Mechanisms affecting intracellular sorting of molecules would therefore feed into the subsequent intercellular strategies. Mobile factors indeed need to reach (and subsequently move away from) PD ([61, 62] as examples). In addition, PD permeability can also be influenced by other organelles (such as chloroplasts and mitochondria) for instance by affecting reactive oxygen species levels in the cell ([63, 64] as examples). The ultimate effect of this is likely largely callose dependent. A more recent example showed that a mitochondrial protein, in this case related to TARGET OF RAPAMYCIN (TOR) metabolic signalling, can also influence permeability [65].

Overall, for cells, flow or “leakage” avoidance may be equally important aspects of permeability control and the relative balance between the two likely depends on the substances moving across, the specific tissues being crossed, the overall developmental status, the environmental context (encompassing both biotic and abiotic factors) and the intracellular partitioning mechanisms. The balance of these mechanisms might altogether enable retention of cell identity while also enabling extensive communication with surrounding cells.

## Molecular ingredients of the PD cake

The nature of PD as membrane-lined pores spanning a wall naturally introduces three molecular structural components of relevance: lipids, proteins (embedded/anchored to a membrane or in the polysaccharide matrix) and polysaccharides. We do not include in this framework proteins that are transiting across PD as part of their cell signalling function but are not resident at PD. We highlight that in our vision these three components are all relevant for PD function as experimental perturbation of any of the three classes can lead to phenotypes.

### Lipids

Lipids are critical components of cellular membranes. They display a diversity of structures and physical properties that have direct consequences on membrane organisation and function, including at PD ([37, 66, 67] for PD and reviewed for general membranes in [68]). Based on their chemical structures, membrane lipids are classified into three main classes: sterols, glycerophospholipids (GPLs) and sphingolipids. Each group is further subdivided into subspecies that present variation in the nature of their polar heads, fatty acid tails (length/saturation) and steryl moieties, creating a vast collection of lipids with distinct physicochemical properties.

**Fig. 2:**
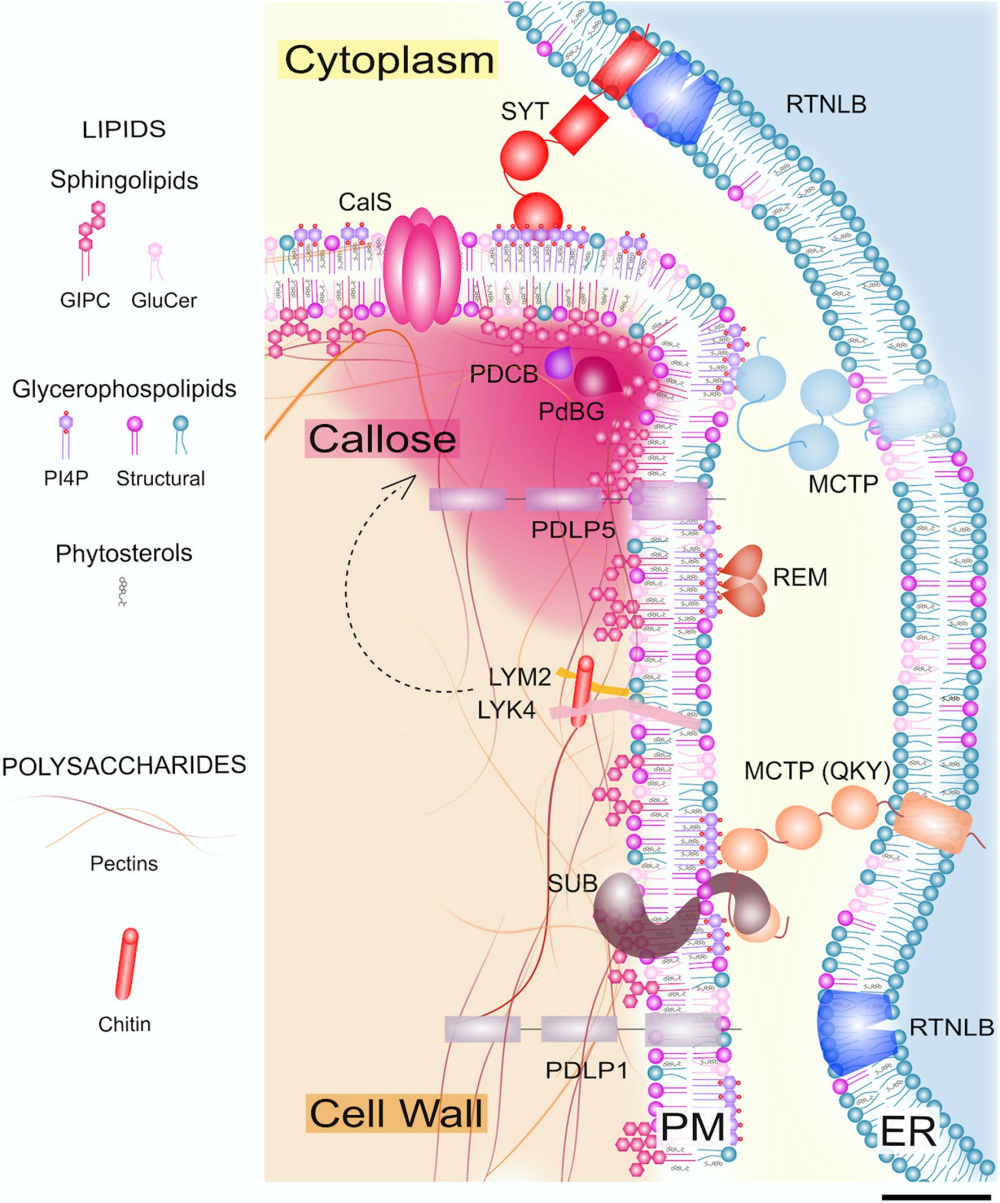
Model of cell wall, lipid and protein distributions and interactions within PD. The upper opening area of a PD is shown in the figure. PD are built up through intimate connections and interactions between the cell wall, the PM and the ER. All these compartments exhibit unique molecular signatures (in terms of lipids, proteins and polysaccharides) at PD. Scale bar: 5 nm. Abbreviations: *CalSs*, callose synthases. *GIPC*, glycosyl inositol phospho ceramides. *GluCer,* glucosylceramide. *LYK4,* LysM-containing receptor-like kinase 4. *LYM2*, LysM domain-containing glycosylphosphatidylinositol-anchored protein 2. *MCTPs*, Arabidopsis multiple C2 domain and transmembrane region proteins. *PdBGs,* plasmodesmal-localized β-1,3-glucanases. *PDCBs,* plasmodesmata callose-binding proteins. *PDLPs,* plasmodesmata-located proteins. *PI4P,* phosphatidylinositol 4-phosphate. *REM*, remorin. *RTNLB*, reticulon-like protein B. *SYT,* synaptotagmin.

Despite cell–cell continuity of the PM across PD, some structural lipids segregate from the bulk PM and are enriched at PD, creating a membrane microdomain with a unique lipid environment. In the first study of the PD membranes [66], the main lipid subspecies for each of the three major lipid classes were conserved between PD and the bulk PM (for instance phosphatidylcholine and phosphatidylethanolamine for the GPLs, glucosylceramide (Glucer) and glycosyl inositol phospho ceramides (GIPCs) for sphingolipids and sitosterol for sterols). However PD-associated GPLs presented a higher saturation level in their fatty acyl chains [66]. The relative proportion between the three lipid classes was also different, with sterols and GIPCs being significantly enriched at PD. In agreement with the lipidome results from Grison, Brocard et al. 2015 [66], another study further characterized the PD sphingolipid backbones and found they were enriched with phytoshinganine (t18:0) long chain bases (LCB) [67]. In the lipid analyses of both studies the ER and PM membranes could not be separated so the PD lipid signatures cannot be unequivocally assigned to either compartment. However, in face of the likely quantitatively larger contribution of the PM to the lipid pool (more extensive surface volume at PD) [66], the low abundance of sterols in the ER (reviewed in [69]) and the modification of sphingolipids in the Golgi apparatus (reviewed in [70]), the lipidomic results might indeed better reflect PM composition. In plants, sphingolipids and sterols are considered as a functional pair, and their interaction has been documented at both chemical and genetic levels ([71] and reviewed in [72]). Sterols present a strong affinity to sphingolipids (and to a lesser extent GPLs) driving, in model membranes and possibly biological membranes, lateral segregation through lipid clustering and leading to the formation of ordered domains (reviewed in [68]) (Fig. 2). A similar process has been suggested to occur at PD [66, 73].

Even within a single membrane compartment, heterogeneity inside the PD pores is likely to exist. For instance, the PD-PM (PM domain lining the PD pores) often adopts positive curvature at the neck region and negative curvature in the central cavity [41], which could drive lateral segregation of lipids with different structural properties [74]. The PM bilayer is also strongly asymmetrical, with GIPC sphingolipids being preferentially located in the outer leaflet while phosphatidylserine and phosphoinositides are  inserted in the inner leaflet (reviewed in [68]). This asymmetrical lipid distribution confers contrasting biochemical properties to the inner and outer leaflets, leading to different functional specificities on cytosolic and extracellular sides [75]. In the context of PD, sterols and certain species of sphingolipids are functionally linked to callose deposition in the outer leaflet [67, 76, 77], while phosphoinositides may help recruit elements for PM-ER tethering at the inner leaflet [78]. The multiple combinations of lipid identities (hence physicochemical properties) and distributions (along the PD-PM, inner/outer leaflet) collectively create the unique membrane properties of the PD pores.

Lastly, the lipid analyses performed so far at PD (and this might also apply to protein and cell wall studies) probably only captured part of the existing diversity. It is fair to speculate that the range of PD structures associated with different tissues, different developmental stages and even different connectivity statuses may very well require lipid changes in their membranes. Likewise, we currently have little understanding as to how specific lipids are clustered and adjusted at PD. Most likely this would operate through local enzymatic activity and targeted vesicular and non-vesicular transport.

### Proteins

Presence of proteins at PD is an implicit corollary to the membranous nature of these structures, as a complete exclusion of transmembrane or lipid-associated proteins would be highly unlikely. However, presence of resident proteins, exclusively localising or displaying increased abundance at PD, is a different expectation that more closely matches the specialised functions of PD.

Proteomic approaches are those that more significantly contributed to capturing the diversity of proteins at PD in a number of species [78–82]. The overlap between the available proteomes and confirmed localisation of some of the detected proteins well substantiate an actual local enrichment of specific sets of proteins at PD. In Arabidopsis, the most recent and curated protein list was provided by Brault, Petit et al., 2019 [78]. The list of 115 proteins well reflects the structural and functional diversity of PD. Cell wall functions are well represented with around twenty members: from enzymes involved in direct callose turnover (such as CALLOSE SYNTHASES, CalSs, and degrading enzymes, PLASMODESMATAL-LOCALIZED β-1,3 GLUCANASES, PdBGs, [83, 84] for experimental validation) (Fig. 2), to those binding to wall components or those modifying other polysaccharides (cellulose, pectin and xyloglucans). We know significantly less about the latter class compared to the former. Proteins with the potential to affect lipids also seem to be present, such as ceramidases, phospholipases, lipases, acyl-esterases, phosphodiesterase, phosphatases. However, confirmed localisations and functional evidence for these at PD is altogether lacking. Proteins that might contribute to the structure and function of PD are also present with for instance several members of the MULTIPLE C2 DOMAIN AND TRANSMEMBRANE REGION PROTEINS (MCTPs) [58, 78, 85], a family sitting at the boundary between the desmotubule and the PM (Fig. 2), which will be discussed in more detail in later sections.

In our description so far, we have narrowly ascribed the function of PD to the direct translocation of molecules. This is, however, incorrect as these structures also operate themselves as signalling hubs. This is the intracrine-like aspect of PD we had hinted at in the introduction. Presence of resident receptor-like proteins at PD, presumably at the PM, is well supported by proteomic studies [78–81]. Functional studies validated the presence of STRUBBELIG/SCRAMBLED (SUB/SCM), a receptor-like pseudo-kinase involved in tissue patterning and morphogenesis [85, 86]; LYSIN MOTIF DOMAIN CONTAINING GLYCOSYLPHOSPHATIDYLINOSITOL ANCHORED PROTEIN 2 (LYM2) receptor-like protein, playing a role in fungal pathogen perception [87]; in association with the other RLKs shown in [88]), and the cysteine-rich receptor-like protein PD-LOCATED PROTEIN5 (PDLP5), important for innate immune responses [89] (Fig. 2). More recent studies even provided examples of kinases dynamically re-localising to PD in response to biotic [88] or abiotic [76, 90] stimuli. CLAVATA1 (CLV1) and ARABIDOPSIS CRINKLY4 (ACR4), form a receptor kinase complex, which localizes at PD, and is important for root meristem maintenance and stem cell signalling [91]. The specific reason for the enrichment at PD of stem cell signalling receptors remains partially unclear. A number of biological processes in meristems do indeed rely on non-cell autonomous signalling ([9] as an example). Presence of these receptors could naturally relate to the positioning of PD at the junction between cells (and indeed some of the downstream signalling effects likely operate to limit PD conductivity, see [87] as an example) but also it might be due to the close contact of various cellular compartments and components (ER-PM-wall) at PD, facilitating coordinated responses. While the ability to perceive external stimuli would be advantageously spread across the entire membrane of a plant cell (detection across a larger surface area), differential and targeted responses across the cell might be subsequently required. This has for instance been shown for the fungal elicitor chitin [88].

So far, most of the listed proteins at PD reside in the PM (as examples [84, 89, 91–94]). Only a handful are embedded in the ER: Calnexins [19], the reticulon-like family [95] and the multiple C2 and transmembrane region protein family [58, 78, 85] (Fig. 2). The latter family actually ends up spanning the cytoplasmic sleeve [78]. Members of the calreticulin family have also been reported as resident in the ER lumen [96, 97]. Overall, we know very little about the function of desmotubule and associated proteins in the context of PD functionality.

Lastly, cytoskeleton related proteins (actin, myosin, tropomyosin, formins, Arabidopsis networked superfamily of actin binding proteins and other actin related proteins) have been localised to PD by immunological and functional studies (reviewed in [98] with more recent additions in [99, 100]). From this list, actin clearly seems to play a role at—and most likely within—PD. Conversely, this does not seem to be the case for microtubules (reviewed in [98]). Actin microfilament fibers might not fit within PD in their traditional conformation (discussed in [101], arguing against models like the one in [102]) so the specific roles of the cytoskeleton and its organisation at PD are, to this day, a debated topic. Formins are suggested to anchor actin filaments to PD, PM and possibly cell wall [100, 103]. Cytoskeletal inhibitor treatments reduced, increased or left transport unaltered across PD depending on species and tissues being studied (reviewed in [98]). The ultrastructure of PD in columella and suspension cells was recently shown to be unaffected by actin inhibitory drugs [41]. This was relevant as the spokes between PM-ER (first reported in [104]) were at some stages suggested to be cytoskeletal in nature ( [102] as an example). This idea has been superseded by the concept of PD as membrane contact sites (reviewed in [48]).

### Cell wall polysaccharides

Plant cells are encased by rigid cell walls performing both structural and developmental functions (reviewed in an evolutionary context in [105]). PD span the wall separating two neighboring cells and, similar to the desmotubule and PD-PM, the wall around PD seem to also possess unique molecular signatures, in particular in terms of wall polysaccharides. We know much less about proteins that might also localise in the wall matrix (some examples are reviewed in [106]). In this section we highlight in particular the biophysical properties that such composition might confer.

A more marked presence of β-(1 → 3)-glucan, commonly known as callose, at the PD wall is well established ([107, 108] as examples). The levels of this polysaccharide inversely correlate with PD permeability, as hinted in the introduction (Fig. 1c). However, in addition to a direct role in modulating PD aperture, callose presence has been reported to reduce stiffness and increase the elastic properties of cellulose hydrogels [109]. It remains to be determined if these physiochemical properties also apply to the in-planta wall.

A developmental correlation between callose deposition and cessation of cell wall thickening has also been reported in modified forms of PD important for long distance transport [110, 111]. Cell wall thickness would determine the length of the path molecules need to cross before entering the neighbouring cell. A reduction in cellulose content seems indeed characteristic of areas rich in PD—so called pitfields [43]. This might underpin the thinner cell walls observed at PD [42] for a new quantitative visualisation of this aspect). Cell wall thickness is also now starting to be integrated in complex cell–cell permeability models [44].

In addition to callose, differential abundances of pectin polysaccharides have been noted using specific antibodies. Homogalacturonans are abundant at PD clusters, although also present more broadly [112–114]. (1 → 5)-α-arabinan containing pectins are enriched in the cell walls surrounding PD clusters [43, 112] while (1 → 4)-β-galactan containing ones are excluded from those areas [112, 113]. Overall, pectins form an interlinked gel-like matrix that—supposedly—might be more amenable to dynamic modifications of PD aperture, in comparison to stiffer cellulose microfibrils, although models for cell wall structure are an evolving topic (discussed for instance in [115]). The specific types of pectins detected at PD might then impart additional mechanical properties: arabinans containing pectins have for instance been associated with cell wall flexibility [116] while galactan containing ones to stiffness [117]. However, PD specific research in this regard remains to be seen.

The specificity of the cell wall environment at PD (or modulation of the same) might be co-opted by invading viruses to target (and favour) their own spread. Interactions with pectin-modifying factors were for example shown to be important for viral movement protein trafficking [118].

Overall, polysaccharide signatures might also relate to PD formation and elaboration. Indeed, while primary PD are generated by the encapsulation of ER strains in the cell wall septum formed during cell division, secondary de-novo PD formation in elongating cells likely requires some form of cell wall modification. Cell wall modifications are also likely to occur when the morphology of existing PD increases in complexity (reviewed in [119]). However, whether a specific cell wall composition and associated biophysical properties are a prerequisite for these processes or an effect of the same is unclear. In most cases we cannot forecast the positioning of PD to assay the cell wall in advance, despite some spatial rules having been suggested [43, 120]. In principle, mechanisms to generate cell wall micro-domains in plants do exist. These tend to operate at the level of the cytoskeleton, via membrane-anchored proteins that locally promote assembly or dis-assembly of microtubules in particular [120, 121]. Cellulose synthase enzymes travel along the microtubules and determine the positioning of the fibrils on the outside of the cell [122]. Ultimately, these molecular mechanisms were shown to regulate the positioning and size of cell wall pits (areas devoid of secondary cell wall deposition) in xylem cells (reviewed in [123]). Whether similar mechanisms might be at play at PD is unknown, yet a fascinating prospect.

This last example is useful to highlight the actual interconnection between lipids, proteins and polysaccharides. We will explore this in the context of PD in the next section.

## Mixing recipes and molecular interactions

We have so far presented for simplicity the molecular components of PD in isolation and highlighted the potential importance of each of them in some contexts. We now want to stress how these three components are, however, interlinked and together determine PD function.

The first hint of this comes from the proteomic approaches to study PD. Although the goal of these approaches is to enrich for a PD fraction while avoiding cell wall and organelle contamination (besides the desmotubular ER and proximal cell wall themselves) (reviewed in [124]), this is never fully achieved as PD can never be separated from the cell wall fraction itself. They have been “baked” together in an undissolvable bond. The relative enrichment for PD proteins versus general cell wall protein relies on a careful level of enzymatic cell wall digestion on the extracted fraction [125]. In some studies what is termed a PD fraction is actually the full cell wall fraction [126]. The recalcitrance of the membranous parts of the PD to separate from the cell wall is likely to reflect direct binding between the two components. The sphingolipid GIPCs, which sit in the outer leaflet of the PM were for instance found to be directly boron bridged to pectins in Rosa cultured cells [127] (Fig. 2). Whether this applies to PD, remains to be established. Changes in sphingolipid levels in biosynthetic mutants seemed to correlate with thicker cell walls at PD [42]. Some PD membrane proteins (besides those directly involved in cell wall biosynthesis or modification) also have polysaccharide binding properties. PLASMODESMATA CALLOSE-BINDING PROTEINS (PDCBs), which are inserted in the outer leaflet of the PM and thereby face the extracellular space [92] (Fig. 2), possess an X8 domain known to bind carbohydrates and more specifically callose [128]. PDLPs’ extracellular domains have structural homology to fungal lectins, also capable of binding carbohydrates [129] (Fig. 2). Interestingly, changes in cell wall have been shown to affect the mobility and nanocluster size of proteins in the PM membrane (albeit not at PD) ([130] via protoplasting; [131] via treatment with cellulose or pectin inhibitors). A similar scenario might be at play at PD.

PD, when they undergo harsh mechanical shredding during isolation or plasmolysis, still retain the ER strand inside, highlighting a tight connection also between lipid membranes [66, 132]. PD proteins indeed tightly engage with local lipids. Glycosylphosphatidylinositol (GPI) lipid anchors serve as minimal sorting signals for preferential insertion in the PM outer leaflet of PD [133]. Proteins can also be directly recruited through their lipid-binding modules. PDLP5 targeting to PD seems to require an interaction between its transmembrane domain and phytoshinganine (t18:0) based sphingolipids, enriched at PD [67]. Interestingly, PDLP1, another PD protein from the same family did not show direct *in-vitro* binding, suggesting that specificity might reside in the structure and sequence of the transmembrane domains (TMs). In a similar manner, MCTP4 PD enrichment may partially rely on their phosphoinositide-binding C2 domains [78] (Fig. 2). Overall, different PD proteins may utilize different lipid species for targeting to different subdomains within PD.

Aside from direct lipid binding, proteins can also recognize membrane properties (such as thickness and curvature) to facilitate their enrichment at specific areas of the cellular membrane (reviewed in [68]) which may also apply to PD. Integral membrane proteins are, for instance, sensitive to local membrane thickness and lipid packing order and consequently segregated into subdomains [75, 134]. PD membranes, as mentioned, are enriched with sterols and very long chain saturated fatty acid—containing lipid species, which presumably make them thicker compared to the bulk PM. Sorting based on transmembrane domain length might therefore be relevant at PD. Indeed, bioinformatic analyses of PD membrane proteins seemed to show on average larger TMs [79]; however, direct physical measurements of membrane thickness [135] are, to this day, not available for PD.

On a different note, biological membranes are by nature heterogeneous. This heterogeneity is caused by specific lipid-lipid but also lipid-protein interactions which altogether result in the formation of microdomains differing in composition and function ([136] as example). This has been observed at PD as well [66, 73] (Fig. 2). For example, sterol-sphingolipid interactions may facilitate lipid nanodomain (also called “rafts”) formation in the PM outer leaflet and recruit GPI-anchored proteins to the PD entry region [66]. Highly ordered nanodomains mediated by sterols and PI4P in the PD-PM inner leaflet host Remorins (REMs). These proteins can locally affect lipid order and influence nanodomain expansion [73, 136]. The relative size and lifetime of PD lipid domains may further determine the repertoire of resident proteins and how these proteins function. Protein mutants and lipid treatments can indeed phenocopy each other (an example in [78] for MCTP mutants and PI4P lipids). However, it would be an oversimplification to largely categorize PD-PM into “raft” and “non-raft” domains; other types of microdomains initiated by specific lipid-protein pairs almost certainly exist. For instance tetraspanin proteins have been detected at PD and other domains [80, 137] and these are known to affect microdomains in other organisms (reviewed in [138]).

Besides the direct physical binding mentioned before, interactions or mutual effects between lipids and cell wall components have also been described, mostly operating via the specific proteins recruited to the PD microdomains. Treatments with sterol inhibitors resulted in altered callose levels at PD and correlated with miss-localisation of PDCB1 protein and callose degrading enzymes PdBGs [66]. In a genetic context, a similar effect was observed in a sterol carrier gene mutant; this time with effects on the transcription of PdBGs and potentially PD structure [77]. Interactions between components can also be elicited by external factors. For instance, changes in lipids brought about by phospholipase enzymes (themselves activated by osmotic-stress) were shown to influence the relocalisation of the CYS-RICH RECEPTOR-LIKE KINASE 2 (CKR2) to PD, which in turn interacted with a callose synthase (CalS1) and led to closure of PD [90].

This set of examples show how lipid, protein and polysaccharide can all be affected by a change in one of these elements.

## Cake structural tiers generated

We conclude this review by emphasizing how these ingredient mixtures ultimately lead to clear PD domains in terms of structure and functional properties. Indeed, as we mentioned in the introduction, PD present elaborate architectures: they contain a central desmotubule (ER) core, surrounded by a cytoplasmic sleeve, in turn delimited by a PM bilayer, itself ultimately surrounded by the cell wall. These four domains are not freestanding: extensive interactions between them exist and are mediated by the molecular players previously described. Functional and dynamic interactions between these different compartments are indeed fundamental for PD function.

### Cytoplasmic sleeve, ER-PM contacts and the regulation of symplastic transport

The cytoplasmic sleeve is regarded as the main route of transport within PD, and a direct correlation between the size of the sleeve and its transport capacity is assumed in current canonical descriptions (see for instance [139]). The cell wall polysaccharide callose, arguably the only well known regulator of PD permeability, is believed to push the PM and ER closer together, reducing the functional sleeve space and hence transport (Figs. 1c and. 2). Enlarged callose collars at PD openings have indeed been observed in EM images [140]. We previously mentioned several examples of callose action and induction in biological contexts. However, mechanistically, how such an extensive change in the cell wall is perceived and accommodated by the PD membranes and transduced to the cytoplasmic sleeve remains unresolved. However, changes in the cell wall may also trigger relevant modifications in the composition or physical properties of the PD membranes.

This direct relationship between cytoplasmic sleeve size and transport was recently challenged by novel structural descriptions of PD. PD populations, especially those in cell types with specialised functions [12] are structurally heterogeneous and diverse. Electron microscopy (for example [30]) and some protein markers ([39] as an example) had enabled classifications based on volume traits, such as number of channels, shape and cavities (overall reviewed in [141]). The use of electron tomography in recent years has put more emphasis on the membrane contact sites nature of PD and it introduced a type classification based on the more subtle degree of apposition between the PM and ER membranes. Newly formed Type I PD display a remarkably close apposition between the two membranes leaving a tiny gap (2–3 nm) filled with electron dense material. Type II PD, however, have the classic conformation of a clear sleeve separating the two [41]. A developmental progression from Type I to Type II was suggested based on the age of the cells, pointing towards an “opening” and modification of the former into the latter [41]. This progression was also correlated with PD protein population changes [78].

Close membranes appositions—such as those observed at PD (in both types)—could require active tethering by proteins (reviewed in [142]). MCTP protein family members have been suggested to perform such a role within PD [78] and, in addition or as a consequence of that, influence both targeted and non-targeted transport (as examples [58, 78, 143, 144]) (Fig. 2). Synaptotagmins family members also act as ER-PM tethers and may have a more significant contribution in the immediate proximities of PD and in particular contexts such as viral infection [145, 146] (Fig. 2). The overall linkage of the ER to PM via the tethers might also be important for rapidly regulating PD conductivity during osmotic shock. A pressure induced sliding of the ER against the openings of the PD pore was recently suggested as a mechanism for closure [147]. The model tries to resolve decades long evidence for pressure regulation of PD (for instance [148]).

Modification of the tether populations, in terms of protein types or conformations of the same (likely also in combination with the underlying lipids in the membranes and possibly in a calcium dependant manner) could regulate the size and conductive properties of the cytoplasmic sleeve and possibly affect transport of substances. The minimal sleeve size would be at least the physical space of the protein tethers themselves (as exemplified in the figures of [149]). How sleeve modifications would be allowed by the surrounding cell wall, which would require relaxation or some degradation to accommodate the opening sleeve, also remains to be elucidated.

What is important in the context of transport is that type I PD, counterintuitively, considered their reduced sleeve, were found to be competent for transport of macromolecules such as GFP [41] and were consistently found in tissues with high cell–cell permeability [35, 41]. They were also ultimately shown to be more permeable than type II PD [37]. The last point clearly breaks the sleeve size assumption we mentioned earlier. How mechanistically transport might occur within such crowded and limited space remains unclear and resolving this will be essential.

### The PM influences the cytoplasmic sleeve and the cell wall environment

The last paper we mentioned [37] highlighted another important point, in this case related to the lipid membranes: sphingolipid biosynthesis (in particular of those species containing very long chain fatty acids) controlled the type transition at the phloem pole pericycle (PPP)—endodermis (EN) interface in the root. Sphingolipids might therefore be among the lipid classes recruiting protein factors important for sleeve regulation at PD. Modifications of the PPP-EN cell wall might also relate to these sphingolipids as differences were detected between mutant and wild-type backgrounds [42], once again further linking the ingredients of the PD cake.

The role of lipids in shaping PD structure has also been suggested in Huang, Sun, Ma et al., [73]. A mechanism was proposed whereby REM proteins oligomerization, induced by salicylic acid (SA) treatment, increases the order level of the lipid bilayer and this, through a still unknown mechanism, leads to PD closure. The mechanism was suggested to be (at least partially) callose-independent and, therefore, act in parallel to the known callose dependent mechanism of SA induced PD closure [89]. REMs are known PM nanodomain proteins [150] and, so far, group1 REMs have been correlated to callose regulation at PD ([151, 152] as examples). Huang, Sun, Ma et al., [73] found that overexpression of REM reduced dye transport in the root but this effect could be partially rescued with chemical treatments that depleted membrane sterols (high order generating components) but not by treatments impairing callose deposition. The authors proposed that REM misexpression rigidifies the PD-PM and reduces the size of the cytoplasmic sleeve. This is an opposite correlation to the Yan, Yadav et al., [37] paper in terms of sleeve size and transport capacities.

Differences within the same lipid class might also be important. While in [37] the change in sphingolipids did not affect any aspect of callose levels, another study has shown that mutations in two other sphingolipid modifying genes conversely lead to the direct recruitment of PDLP5, increased callose deposition, reduced PD permeability in leaves and enhanced resistance to pathogens [67]. A direct comparison between the two studies is difficult but the lipid changes in the two are, however, clearly different: the mutation of interest in Yan, Yadav et al., [36] reduces most sphingolipid classes while the mutations in Liu et al., [67] conversely increase them. Specific lipid alterations, with likely compensatory effects from the other lipids in PD membranes, might therefore lead to recruitment of different PD proteins, depending on the specificity of the latter. Different tissues and their specificities might also contribute to differences between the studies. It is also possible that, since certain sphingolipid species carry signalling function (reviewed in [153]), the change elicited in Liu et al., [67] actually leads to a broader immune response involving callose (rather than PD structural changes). In future work, it will be interesting to understand how callose deposition might affect the local PD-PM organisation/properties and impact on the ER-PM contact sites within PD.

### The desmotubule and its structural and transport capabilities

The instinctive focus on the cytoplasmic sleeve is built on the idea that PD mainly support cytosolic trafficking. However, another structural domain within PD is a constricted form of the ER (in close contact with the PM). This has been observed since the earliest identification of these channels in plants [154].

The presence of the ER inside PD that originated from cell division (termed primary PD) has largely been taken as a side effect of ER strands trapping in the forming cell plate [17]. This integration of ER tubules into the forming cell plate [155] is well contrasted with active ER clearance and abscission during cytokinesis in yeast and mammalian cells (reviewed in [156]). The reason for such drastic differences in ER network organization during cell division remains largely elusive. In land plants, the ER seems to be a prerequisite for building up PD pores, as fenestrae in the cell plate only develop into PD when the ER pre-occupies such positions [155] and no PD without a central ER have ever been observed. The presence of the ER inside PD that are formed independently of cytokinesis (termed secondary PD) [157] is an additional argument against the idea that the ER is just being passively trapped inside PD. The formation of PD (primary and secondary alike) is likely to require tethering between the PM and the ER [41, 145, 157]. To further support such point, genetic disruption of PD tethers in maize, namely mutating the CARBOHYDRATE PARTITIONING DEFECTIVE 33 protein (ortholog of members of the MCTP family in Arabidopsis), results in a significant reduction in PD at the companion cell—sieve element interface in leaf veins [143].

Researchers seldomly consider the impact the desmotubule has on PD function and how the latter would be affected in absence of the same. Some PD analogous structures in algae lack a desmotubule (reviewed in [6]), hinting that presence of ER is not an absolute requirement for connectivity, although, as suggested by Park et al., [147], deformation of the desmotubule/ER might be important for regulating trafficking through the cytoplasmic sleeve. Intriguingly, in comparison to other membranous channels described across the eukaryotic kingdom, only plant PD exhibit an intracellular organelle as an integral part of the structure (reviewed in [6]). This singularity of PD highlights the value of investigating the function of the ER. The desmotubule likely performs some selectively advantageous purpose making such membrane a pivotal player, rather than an entrapped victim.

At a local scale, close contact between the opposing membranes from two organelles might facilitate non-vesicular transport and intraorganellar communication (reviewed in [158]). Within PD, it is rational to speculate that ER takes part in establishing the unique lipid environment of PD by recruiting proteins that modify the lipids or directly transfer lipids between two membranes. Such actions on PM lipid modification could have a direct impact on membrane shaping, protein recrutement, organisation and even function (via conductive and structural properties of PD).

From a geometric and fluid dynamics point of view, having a ER tubule inside a PM channel significantly increases transport capacity while maintaining size selectivity, as recently suggested by mathematical modeling [44]. A PD with a desmotubule (tubule within a tube structure), in theory, provides more transport volume than a cylinder channel without a desmotubule. This can be explained by the fact that the cytosolic cross-sectional area of a PD with desmotubule is much larger than the cross-section of a cylindrical channel with the same theoretical SEL (which corresponds to the maximum particle radius that fits in the channel). Hence, with a desmotubule radius of 8 nm and a maximum particle radius of 2 nm, 20 cylindrical channels would be needed to match one PD with a desmotubule. The presence of a desmotubule also reduces the surface area in proximity to the wall and hence steric hindrance and viscous drag. As particle centers cannot come closer to a surface that their own radius (steric hindrance), the available area for a particle size of ½ SEL would be 25% in a cylindrical channel and 50% in a channel with desmotubule. Forty cylindrical channels would then be required to match one PD with desmotubule. This difference in transport capacity further increases when considering the hindrance effects from viscous drag along the channel surface (Eva Deinum, personal communication).

Lastly, the desmotubule could work in trans by modulating PD-PM signalling. This was recently suggested at PD for ER protein MCTP15/QUIRKY, which promotes, through direct physical interaction, the stabilisation and signalling of PM localised SUB [85, 144] (Fig. 2).

ER continuity through PD could also serve as a route for intercellular transport, as we briefly discussed in the introduction. Lipid derived signals related to plant immunity have been suggested to propagate between cells along the desmotubule and lipid transfer proteins located in the ER would facilitate this [159]. Diacylglycerol was suggested to diffuse along the ER membranes of PD when microinjected, while sphingolipids could not move along the PM [15, 16]. PD membranes might, therefore,  represent both routes of exchange and isolation points. Transport of native transmembrane proteins along PD membranes remains to be demonstrated but targeted transport has been shown for viral movement proteins (reviewed in [160]). Movement within the desmotubule lumen was experimentally ruled out for proteins the size of GFP or greater [161, 162] although lumenal transport of 10 kDa molecules has been reported in stem epidermal cells [163] and leaf trichome cells [164]. Preferential intercellular movement of zinc ions has also been speculated to occur across the desmotubule in mineral deficiency conditions [165]; evidence for this is, however, lacking. Overall, mechanisms to regulate such flows have not been clarified.

Similar to plants, mammalian and yeast cells share strands of ER between cells during cell division. However, they establish an ER diffusion barrier to prevent uncontrolled flow along the membrane, especially of substances related to ER stresses and aging [166, 167]. Whether this is also the case in plants remains largely unexplored. In addition, it is interesting to point out that, while yeasts and mammalian cells do not establish a barrier for transport within the connected ER lumen, plants clearly do. The extreme physical constriction of the desmotubule seems to largely prevent protein transport [161, 162]. It has been shown that both ER shaping proteins, such as reticulon-like proteins (RTNLB) [95] and ER-PM tethers of the synaptotagmin SYT family [145] contribute to extreme ER constriction at PD (Fig. 2). The mechanisms through which ER constriction is specially executed within PD—and why this may be relevant in the context of plant multicellularity—again remain to be determined.

## The cell wall and apoplastic /symplastic cross talks

Earlier in this review, we highlighted the existence of direct receptor mediated signalling at PD and we mentioned its role on PD permeability. Here, in the context of the structural layers, we want to stress how this offers opportunities at PD for signal integration between the symplast and the apoplast. For the role in the apoplast sensing, one could envisage aspects of paracrine signalling applying to PD.

Some proteins perform a direct relay of an extracellular clue to the inside of the cell. This is for instance the case of LYM2/RLK receptor complex, perceiving fungal chitin in the apoplast and triggering a signalling cascade resulting in PD closure via callose (Fig. 2). LysM receptor-like kinases 4 (LYK4) is one of the kinases that conditionally associates with LYM2 (Fig. 2). The response aims to block the spread of potential pathogen effectors and also regulate movement of endogenous defence signalling molecules [87, 88, 168]. Other proteins highlight more significant integration between the two transport pathways. ACR4 (acting in homomeric complexes and heteromeric ones with CLV1), for instance, detects the secreted CLAVATA3/ESR-RELATED (CLE) 40 peptide in the apoplast of root tips and influences stem cell maintenance [91]. A mechanism was suggested by which a gradient of CLE40 peptide within the root tip would lead to a dose dependent activation of ACR4-CLV1 complexes at PD. The differential intracellular activity of the receptors in the various cell layers would then promote or repress the PD symplastic mobility of unknown stem cell factors (reviewed in [169]). While experimental evidence of this model is not yet available, the mechanism would act as a robust positional system to balance meristem maintenance and differentiation. A potentially similar mechanism could involve the beta glucanase ZERZAUST (ZET). This protein is atypical as it does not seem to actually degrade callose nor to localise to the PM despite its GPI anchor. Conversely, it localises to the cell wall and moves in the apoplast between cell layers of lateral roots [170]. Mutations in this gene result in phenotypes similar to SUB mutants [171]. SUB/SCM is a receptor like kinase partially localising to PD and involved in tissue morphogenesis and patterning [85, 144] likely by affecting the movement of an unknown mobile factor. The interaction between SUB and ZET (if any) is, however, not direct [170]. It is possible to speculate of other cases where signal integration might be happening. For instance, switches between symplastic and apoplastic loading/unloading of molecules, associated with developmental stages or triggered by biotic challenges [27, 172–175], might require some coordination. This could partially occur at PD. Systemic acquired resistance (SAR), one of the long-term plant defence responses to pathogens, might also benefit from signal integration. Some of the signals required to trigger this response indeed travel in the apoplast (salicylic acid) while others move in the symplast (azelaic acid and glycerol-3-phosphate). The latter two have been shown to interact with proteins also localising to PD [159, 175] and SAR has been shown to depend on PD function [175].

## Prospects and future baking endeavours

Over the last decade, remarkable progress has been made in identifying the molecular elements and building blocks of PD pores. While this process has not exactly been a piece of cake—but rather a complex meal to digest—the extent of detail on protein composition of PD we have now accumulated, the characterisation and emerging roles of lipids and the continued relevance of cell wall polysaccharides have all proved to be significant milestones. The novel functionalities of PD as direct receptor-signalling hubs and their capacity to couple symplastic and apoplastic signalling have also added novel functional angles to these structures. Recent work has also questioned well-accepted textbook models, for instance, the direct relationship between ER–PM spacing and the transport capacity of the cytoplasmic sleeve. Overall, accumulating evidence clearly suggests a synergetic action between lipids, proteins and polysaccharides. The multiple cake layers of PD (ER–sleeve–PM–cell wall) are also all fundamentally and functionally intertwined. Only by embracing this close-knit molecular and architectural complexity, the field will be able to further resolve the subtle and hidden flavours of PD.
